# Coherence in cooperative photon emission from indistinguishable quantum emitters

**DOI:** 10.1126/sciadv.abm8171

**Published:** 2022-03-18

**Authors:** Zhe Xian Koong, Moritz Cygorek, Eleanor Scerri, Ted S. Santana, Suk In Park, Jin Dong Song, Erik M. Gauger, Brian D. Gerardot

**Affiliations:** 1 SUPA, Institute of Photonics and Quantum Sciences, Heriot-Watt University, Edinburgh EH14 4AS, UK.; 2 Centro de Cîencias Naturais e Humanas, Universidade Federal do ABC, Santo Andrè, São Paulo 09210-580, Brazil.; 3 Center for Opto-Electronic Materials and Devices Research, Korea Institute of Science and Technology, Seoul 02792, Republic of Korea.

## Abstract

Photon-mediated interactions between atoms can arise via coupling to a common electromagnetic mode or by quantum interference. Here, we probe the role of coherence in cooperative emission arising from two distant but indistinguishable solid-state emitters because of path erasure. The primary signature of cooperative emission, the emergence of “bunching” at zero delay in an intensity correlation experiment, is used to characterize the indistinguishability of the emitters, their dephasing, and the degree of correlation in the joint system that can be coherently controlled. In a stark departure from a pair of uncorrelated emitters, in Hong-Ou-Mandel–type interference measurements, we observe photon statistics from a pair of indistinguishable emitters resembling that of a weak coherent state from an attenuated laser. Our experiments establish techniques to control and characterize cooperative behavior between matter qubits using the full quantum optics toolbox, a key step toward realizing large-scale quantum photonic networks.

## Teaser

Coherent coupling of two solid-state emitters within a diffraction-limited spot demonstrates Poissonian-like photon statistics.

## INTRODUCTION

Cooperative photon emission can arise between quantum emitters because of photon-mediated interaction via a shared electromagnetic mode. This can occur with indistinguishable atoms, or artificial atoms, which emit identical photon wave packets and cannot be spatially distinguished. Atomic indistinguishability leads to entangled multiparticle states referred to as Dicke states (*1*). In ensembles of densely packed atomic or solid-state emitters, Dicke states can yield sub- and superradiant emission with modified temporal, spectral, and directional properties (*2*–*6*). At the few-emitter level, cooperative emission has been observed with emitters positioned closely (interatomic separation Δ < λ, the photon wavelength) in free space (*7*, *8*) or coupled to one-dimensional waveguides (*9*–*14*). Dicke states offer intriguing potential to engineer quantum states for applications in quantum information processing (*15*–*19*) and precision metrology (*20*, *21*).

Atomic correlations can also occur from quantum interference between distant quantum emitters. This is commonly achieved by using a beam splitter to erase the which-path information from two indistinguishable emitters, providing a route to realize scalable quantum networks (*22*–*27*). Similarly, interference in the far field of spatially separated sources of indistinguishable single photons also gives rise to entanglement and Dicke states (*28*–*30*). Compared to the spontaneous emission from a single atom or a group of distinguishable atoms, a signature of the atomic entanglement that underlies cooperative emission is a change in the second-order intensity correlations; for instance, “bunching,” rather than “antibunching,” arises at zero delay in a Hanbury Brown-Twiss (HBT) interferometer (*10*, *12*–*14*, *31*–*33*). While cooperative emission and in particular sub- and superradiance have been extensively explored in both theory and experiment, coherent control of the correlations and the effects of indistinguishability and dephasing have yet to be investigated. Furthermore, higher-order intensity-intensity correlations that characterize the coherence (*34*) of cooperative emission remain unexplored.

Here, we report on the cooperative emission from two proximate semiconductor quantum dots (QDs) that can be tuned into resonance electrically. Collecting the emission using a diffraction-limited focus at Δ/2 leads to the erasure of spatial and spectral distinguishability of the photons, creating emitter entanglement between the two dots, although the separation between them exceeds the wavelength of the emitted photons, Δ > λ. The ability to tune the emitter via an applied bias allows us to contrast the photon statistics of distinguishable to that of indistinguishable emitters. Following the detection of a first photon, the detection probability of the subsequent photon is halved in the former case, because only one emitter remains excited and can contribute to photon emission. By contrast, for cooperative emission from indistinguishable emitters, where both emitters are involved in both photon emission processes, the emission rates for the first and the second photon are identical. As emission with a constant rate defines Poissonian statistics, the second-order correlation function then resembles that of a weak coherent state from an attenuated laser (*35*).

We probe the different statistics of distinguishable versus indistinguishable emission via second-order intensity correlations and with a Hong-Ou-Mandel (HOM)–type interferometer (*36*). For both measurement configurations, we observe increased (zero-delay) correlations and Poisson-like photon statistics under continuous wave (CW) and pulsed driving. Compared to strict resonance fluorescence, we observe a reduced level of correlation using nonresonant excitation (into the phonon sideband, blue-detuned from the zero-phonon line) due to increased emitter dephasing and time jitter of the exciton population. Time-resolved resonance fluorescence of the independent (spectrally detuned) QDs and the correlated indistinguishable QD system reveals identical lifetimes, i.e., we observe no reduction in the emission lifetime, as would be expected for superradiance (*2*). This indicates that the zero-delay peak in the HBT experiment is not a sufficient witness of superradiant emission with collective rate enhancements but is a sensitive probe of cooperative emission. Our work establishes techniques from the quantum optics toolbox to control and characterize collective light-matter interactions.

**Fig. 1.**
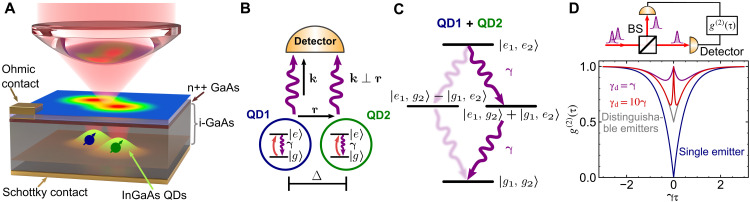
Dicke states and cooperative emission from two indistinguishable quantum emitters. (**A**) A schematic of the sample and spectroscopy setup, showing common-mode optical excitation and collection from two negatively charged InGaAs QDs embedded in an intrinsic (i-) GaAs region of a gate-tunable Schottky diode structure with an Ohmic contact to an n-doped (n++) GaAs layer. (**B**) Detection of photons with wave vector (**k**) emitted from two indistinguishable QDs, spatially separated along (**r**) by Δ and equally coupled to the same driving field, projects the system into the symmetric state (∣*e*_1_, *g*_2_⟩ ***+*** ∣*g*_1_, *e*_2_⟩). The spontaneous emission rate of a single QD is γ. (**C**) Schematic of the two-atom Dicke ladder, showing the bright transition from the doubly excited state (∣*e*_1_, *e*_2_⟩) to the symmetric state and subsequently to ground state (∣*g*_1_, *g*_2_⟩). Transitions via the antisymmetric state (∣*e*_1_, *g*_2_⟩ **−** ∣*g*_1_, *e*_2_⟩, blurred arrows) are not directly monitored. (**D**) Second-order correlation function (*g*^(2)^(τ)) measured using the HBT setup (top) indicates the emergence of the antidip around the zero delay due to cooperative emission from two indistinguishable emitters, each with a pure dephasing rate of γ_d_. BS: 50:50 Beam splitter.

Figure 1A shows the schematic of our experiment: A confocal microscope with a diffraction-limited focus is used for both optical excitation and photon collection from two QDs. Not shown in the schematic is a hemisphere solid immersion lens (SIL), with index of refraction *n* = 2.0, which is placed on top of the sample to increase the collection efficiency from the QDs (*37*). Crucially, the focal spot is optimized to ensure equal excitation and collection from both emitters, erasing the spatial distinguishability of each emitter. This is illustrated graphically in Fig. 1B, in which two identical QDs, each with spontaneous emission rate γ, emit photons with wave vector **k** orthogonal to the separation between the emitters **r**. The indistinguishability of detected photons ensures that, upon measurement of a single photon from a two-emitter system initially in the doubly excited state∣*e*_1_, *e*_2_⟩, the wave function collapses to the maximally entangled Dicke state $\left(1/\sqrt{2}(|e_{1},g_{2}\rangle +|g_{1},e_{2}\rangle )\right)$ (cf. Fig. 1C and Materials and Methods). The entanglement enables both emitters to cooperatively take part in the second photon emission process, which enhances photon coincidences compared to the emission from uncorrelated emitters (*38*). Here, the entanglement is induced by the measurement process. In contrast, entanglement between emitters in the superradiant regime arises from free radiative decay alone (*1*, *2*). Figure 1D shows examples of the second-order correlation function *g*^(2)^(τ) near zero time delay (cf. section S1) for CW driving of (i) a single quantum emitter that exhibits perfect antibunching *g*^(2)^(0) = 0 (blue curve); (ii) two distinguishable quantum emitters that exhibit *g*^(2)^(0) = 0.5 (gray curve); and (iii) two indistinguishable quantum emitters with different dephasing rates γ_d_ = γ and 10γ (purple and red curves, respectively). An instructive example for which an analytic solution is available is the special case of incoherent pumping with equal pump and decay rates γ_p_ = γ, where one obtains a delay-time dependence of cooperative emission of the form

$$g^{\left(2\right)}(\tau )=1-(e^{-2\gamma \tau }-e^{-(2\gamma +\gamma_{d})\tau })/2$$ (1)

This yields the “antidip” at τ = 0 with a width determined by the dephasing γ_d_. As the main effect of dephasing here is to reduce the coherence between states with a single excitation in either emitter and therefore the correlations, the strong dependence of the *g*^(2)^(τ) signal on the dephasing further highlights the importance of interemitter entanglement for the enhancement of photon coincidences.

## RESULTS

### Tuning two near-degenerate QDs into resonance

**Fig. 2.**
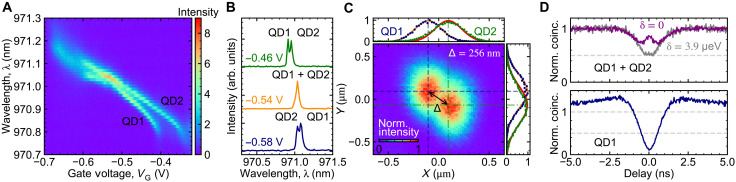
Tuning two near-degenerate QDs into resonance. (**A**) PL spectra (nonresonant excitation) of negatively charged exciton transitions from QD1 and QD2 as a function of applied gate voltage (*V*_G_). (**B**) Individual PL spectra at *V*_G_ = −0.46, −0.54, and −0.58 V. The QDs are tuned into degeneracy at *V*_G_ = −0.54 V. (**C**) Spatial profile of both QDs at *V*_G_ = −0.46 V accounting for the ×2 magnification of the SIL. The Gaussian fits (solid lines) to the experimental data (circles) give the separation of two QDs of Δ = 256 nm. (**D**) Top: Second-order intensity correlation for emissions from both QDs (QD1 + QD2) reveals an antidip around zero time delay for the case at zero detuning (δ = 0) and a dip showing a *g*^(2)^(0) ≈ 0.5 for nonzero detuning (δ = 3.9 μeV). Bottom: Second-order intensity correlation (*g*^(2)^) for emission from QD1 reveals dip at the zero delay, showing a *g*^(2)^(0) ≈ 0.06, signifying single-photon emission.

The sample consists of self-assembled InGaAs QDs embedded in a Schottky diode to control the QD charge state via Coulomb blockade (*38*) and allow a small range of energy tuning via the DC Stark effect (*39*). Details on the device design are found in Materials and Methods. The self-assembly process leads to random spatial positions and an inhomogeneous distribution of QD energies and spontaneous emission lifetimes (γ). We therefore search the sample for the unlikely situation in which two QDs (i) are close enough to each other to optically couple to the same focal point, (ii) have very similar transition energies and γ, and (iii) have different permanent dipole moments such that the QDs can be tuned into resonance with a vertical electric field. We choose to work with negatively charged exciton (*X*^1−^) transitions, which, unlike neutral excitons, lack fine-structure splitting. Figure 2A shows the photoluminescence (PL) (using nonresonant excitation) for our chosen QD pair (*X*^1−^ transitions) as a function of gate bias. The three line cuts at different applied biases in Fig. 2B demonstrate the ability to tune the two QDs to the same emission wavelength using only the applied gate voltage. The resonance is found at *V*_G_ = −0.540 V. Here, λ ≈ 971 nm in free space while inside the GaAs (with index of refraction *n* ≈ 3.67), λ_GaAs_ ≈ 265 nm. Fixing *V*_G_ = −0.460 V (where both QDs are spectrally distinguishable, detuned by δ ≈ 70 μeV), we spatially map the precise locations of the two QDs by scanning the sample position while recording the emission spectra. Assigning the peak at lower (higher) wavelength to be QD1 (QD2), we obtain the spatial profile of each QD, as shown in Fig. 2C. Gaussian fits to the intensity versus scanner position gives the spatial separation of the two QDs: Δ = 256.1 (1) nm. We note that for the two QDs in GaAs, Δ ≈ λ_GaAs_, beyond the expected range for substantial superradiant emission enhancement (*2*). Here, we define the origin (*X*,*Y*) = (0,0) as the optimal spatial position to ensure equal collection from both QDs. For all subsequent experiments, we perform all measurements at this position and use the Stark shifts to render the emitters degenerate or detuned.

To confirm the indistinguishability and cooperative emission of the two QDs when degenerate, we obtain resonance fluorescence under CW driving and measure the second-order correlation function *g*^(2)^(τ) with the HBT interferometer. Figure 2D shows *g*^(2)^(τ) for three scenarios at an excitation power (*P*) of *P*/*P*_sat_ ≈ 0.1, where *P*_sat_ is the excitation power at saturation (*40*). When the QDs are detuned and only QD1 is addressed resonantly, *g*^(2)^(0) → 0, indicating single-photon emission. To equally drive the QDs when they are detuned (δ ≈ 3.9 μeV), we excite both emitters slightly off resonance with a laser detuned by ±δ/2 and obtain *g*^(2)^(0) → 0.5, as expected for two distinguishable emitters emitting uncorrelated single photons. Last, *g*^(2)^(0) for resonance fluorescence from the two degenerate QDs (δ = 0) reveals the emergence of a zero-delay antidip, in agreement with previous reports for superradiant emission (*10*, *12*, *13*). This signature confirms cooperative emission from indistinguishable QDs. We note that the slight bunching in coincidences away from the zero delay for the single-emitter case is due to spectral fluctuations. This is not observable for the two-indistinguishable-emitters case as their spectral fluctuations are not fully correlated. We fit the experimental data to an analytical equation (c.f. Eq. 1), convolved with the Gaussian instrument response function [full width at half maximum (FWHM) = 0.240 ns] and obtain a decay time of (2γ)^−1^ = 0.880 (22) ns, dephasing time of $\gamma_{d}^{-1}=0.199\;(10)\;\text{ns}$ and hence a coherence time of (2γ + γ_d_)^−1^ = 0.162 (9) ns, indicating the 1/*e* width of the antidip. While we observe *g*^(2)^(0) ≈ 0.87 from the data/fit, upon deconvolution, we obtain *g*^(2)^(0) ≈ 1, as expected from collective emission from two indistinguishable emitters.

Last, as we increase the driving strength, we observe Rabi oscillations in the *g*^(2)^(τ) coincidence histogram along with the zero-delay antidip. These results, depicted in section S3, show excellent agreement with simulated data produced by our model in section S1.

### Coherent control of cooperative emission

**Fig. 3.**
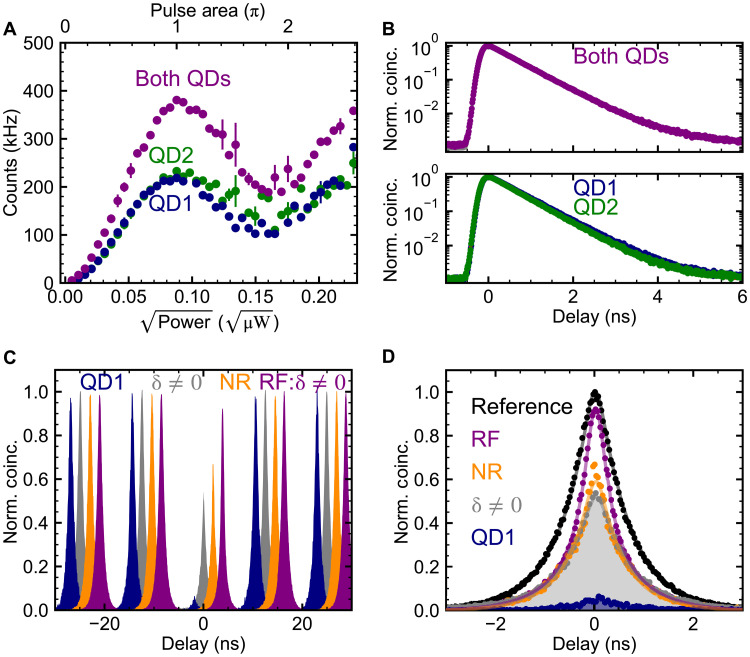
Coherent control of two QDs. (**A**) Emission intensity as a function of excitation power and pulse area from QD1 (blue), QD2 (green), and both dots when degenerate (purple) under pulsed resonance fluorescence, RF. (**B**) Time-resolved emission profiles of the emission from the degenerate QDs (top) and individual emitters (bottom, QD1 and QD2) exhibit similar lifetimes of *T*_1_ = 0.643 (1) and 0.668 (1),0.635 (1) ns, respectively. The pulse area is π/2 for each measurement. (**C**) Second-order intensity correlation of the emission under resonant excitation [QD1 (blue) and both QDs at nonzero detuning (δ ≠ 0, gray) and zero detuning (δ = 0, RF, purple)] and nonresonant excitation for the degenerate QDs (NR, orange). (**D**) Zoom-in of the zero-delay peaks in (C). The data for “Reference” are the side peak at 12.44 ns for the RF case. Measurements for RF, δ ≠ 0, and QD1 are taken at the pulse area of π/2, while measurement for NR is taken at the saturation power.

To manipulate the Dicke ladder populations and probe the transient emission behavior, we perform resonance fluorescence and time-resolved measurements using a pulsed laser. Figure 3A shows coherent Rabi oscillations in the detected emission count rate as a function of pulse area for each emitter (when detuned) and for both QDs when degenerate. Each QD exhibits the same excitation power to Rabi frequency conversion and similar count rates, while the degenerate case yields the identical power to Rabi frequency conversion and the measured count rates are a sum of the individual QD count rates. Fixing the excitation power corresponding to a pulse area of π/2, we measure the temporal emission profile for each case with a time-resolved resonance fluorescence measurement, as illustrated in Fig. 3B. Here, we observe nearly identical temporal profiles and lifetimes in each case: *T*_1_ = 0.668 (1),0.635 (1), and 0.643 (1) ns for emission from QD1, QD2, and both QDs when degenerate, respectively. These results confirm that the temporal properties of the cooperative emission process are unchanged from the independent QDs; superradiance with a modified temporal profile is not observed, as expected for QDs with Δ ≈ λ_GaAs_.

Figure 3C presents *g*^(2)^(τ) for resonance fluorescence at a pulse area of π/2 for four representative cases, with each dataset offset by a few nanoseconds on the *x* axis for clarity. A zoom-in around zero delay (without *x*-axis offset) is shown in Fig. 3D. Overall, the pulsed resonance fluorescence behavior is analogous to that obtained under CW driving. At zero delay, we observe *g*^(2)^(0) → 0, *g*^(2)^(0) ≈ 0.5, and *g*^(2)^(0) → 1 for an individual emitter (QD1), distinguishable emitters (δ ≠ 0), and indistinguishable QDs (resonance fluorescence, RF), respectively. For the individual-emitter case (QD1), the two QDs are detuned by ∼100 μeV. Integrating the coincidences within a 10-ns window around the zero delay, we obtain $g_{\Delta \tau =10\;\text{ns}}^{\left(2\right)}(0)=0.051\;(1)$, 0.510 (2), and 0.672 (1) for each respective case. Restricting the integration window to 0.3 ns, while the $g_{\Delta \tau =0.3\;\text{ns}}^{\left(2\right)}(0)$ values for emission from QD1 and distinguishable QDs remain the same, we instead obtain a much higher $g_{\Delta \tau =0.3\;\text{ns}}^{\left(2\right)}(0)=0.904\;(5)$ for two indistinguishable QDs, approaching the theoretical maximum of 1. Comparison of these two integration windows of $g_{\Delta \tau }^{\left(2\right)}(0)$ for the degenerate QDs suggests that the cooperative emission is sensitive to the degree of indistinguishability or dephasing between the two emitters. This effect is easily visualized by comparing the *g*^(2)^(0) peak for the indistinguishable QD (RF) to a reference peak (e.g., at τ = 12.44 ns, black plot), as shown in Fig. 3D. Here, we observe narrowing of the zero-delay peak compared to the side peaks at 12.44 ns. We then fit the experimental data (zero-delay peak) with Eq. 2 (convolved with the Gaussian instrument response function with FWHM = 0.240 ns) to extract the dephasing parameter γ_d_

$$g_{\text{Pulsed}}^{\left(2\right)}(\tau )=(e^{-\gamma \tau }+e^{-(\gamma +\gamma_{d})\tau })/2$$ (2)

Using the measured lifetime, γ^−1^ = 0.643 (1) ns, we extract a dephasing time of $\gamma_{d}^{-1}=0.280\;(7)\;\text{ns}$ and hence a coherence time of (γ + γ_d_)^−1^ = 0.195 (3) ns from the fit. By contrast, in the absence of dephasing (γ_d_ = 0), the width of the zero-delay peak (given by Eq. 2) is the same as that of the side peaks (equal to the radiative lifetime γ^−1^), resulting in $g_{\Delta \tau }^{\left(2\right)}(0)=1$ for any integration time Δτ. This supports the interpretation that the degradation in $g_{\Delta \tau }^{\left(2\right)}(0)$ at larger Δτ is related to emitter dephasing.

To experimentally demonstrate that the $g_{\Delta \tau }^{\left(2\right)}(0)$ value for cooperative emission is sensitive to dephasing, we excite the degenerate QDs using pulsed nonresonant, phonon-assisted excitation [NR, orange plot in Fig. 3C] and observe reduced bunching compared to strict resonance fluorescence: $g_{\Delta \tau =0.3\;\text{ns}}^{\left(2\right)}(0)=0.624\;(7)$ and $g_{\Delta \tau =10\;\text{ns}}^{\left(2\right)}(0)=0.556\;(4)$ within 0.3- and 10-ns integration windows, respectively. In addition, fitting the data to Eq. 2 results in a coherence time of 0.043 (2) ns, significantly shorter than that observed with coherent driving. We ascribe this reduction to reduced indistinguishability between the two QDs when using incoherent excitation, likely due to decoherence from the phonon-assisted transitions as well as increased charge noise and time jitter. Additional detailed spectroscopy results (power dependence and detuning dependence) under pulsed coherent and incoherent excitation are included in sections S4 and S5, respectively. In the case of coherent driving of two indistinguishable QDs, we observe an oscillating $g_{\Delta \tau }^{\left(2\right)}(0)$ value as we vary the excitation power of the resonant pulse, demonstrating the ability to coherently manipulate the populations on the Dicke ladder.

Last, we note that the bunching around zero delay demonstrates entanglement between the two QDs. In the absence of detector jitter, the limit *g*^(2)^(τ → 0) indicates the degree of the instantaneous entanglement immediately after the first-photon detection event as it is related to the occupation of the Dicke state (cf. Materials and Methods or section S2). However, in a realistic experimental setting, this limit is largely determined by finite detection jitter [with *g*^(2)^(0) = 1 for perfect detectors] and thus is not a reliable metric. Therefore, we consider the integral of the entire zero-delay peak, relative to the adjacent uncorrelated side peaks, as a more representative indicator of the degree of entanglement. This quantity $g_{\Delta \tau }^{\left(2\right)}$ has the further advantage of being resilient to details of emitter dephasing that determine *g*^(2)^(τ → 0). The degradation in $g_{\Delta \tau }^{\left(2\right)}$ due to the increase in the dephasing under nonresonant excitation (at saturation) results in a lower value of $g_{\Delta \tau =10\;\text{ns}}^{\left(2\right)}\approx 0.56$ compared to that under resonant excitation $g_{\Delta \tau =10\;\text{ns}}^{\left(2\right)}\approx 0.67$ (at a pulse area of π/2). Correcting for both the laser leakage and the detection jitter, we obtain the associated entanglement fidelities (to the maximally entangled symmetric Dicke state), upon a detection of a single photon for both cases to be F ≈ 0.56 and F ≈ 0.60, respectively. Please refer to section S6 for a detailed discussion.

### Higher-order intensity correlations of cooperative emission

Next, we probe the coherence of the cooperative emission by interfering subsequently emitted photons in a conventional HOM-type interferometer setup. The measurement setup consists of an unbalanced Mach-Zehnder interferometer with a delay to match the temporal separation of the excitation pulses, i.e., Δ*T* = 12.44 ns. To quantify the interference visibility *V*_HOM_(τ), we define

$$V_{\text{HOM}}(\tau )=1-g_{∥}^{\left(2\right)}(\tau )/g_{⊥}^{\left(2\right)}(\tau )$$ (3)

where in the case of single-photon input, *V*_HOM_(0) is the ratio of coincidences at zero delay when photons in the two paths of the interferometer are rendered indistinguishable ($g_{∥}^{\left(2\right)}(0)$) and distinguishable ($g_{⊥}^{\left(2\right)}(0)$) in polarization. This polarization control of the input photons is achieved by rotating the half-wave plate in one arm of the interferometer. While V_HOM_(τ) gives the single-photon indistinguishability for a single-photon indistinguishability for a single-photon input, it provides a measure of the degree of coherence (*34*) of the photons from both emitters.

**Fig. 4.**
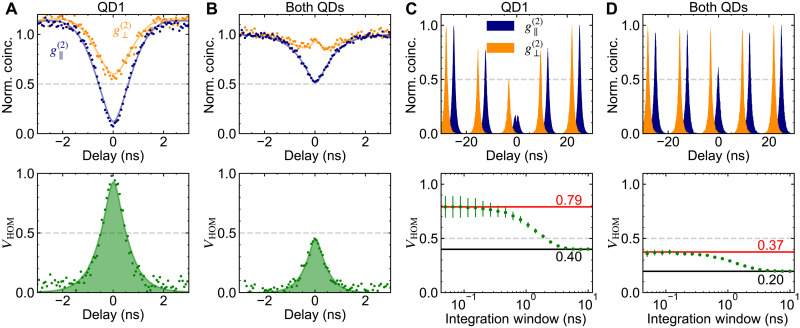
HOM-type interferometry of cooperative emission. (**A** and **B**) Normalized photon coincidences for the case where input photons from only QD1 (A) and from both QDs (B) are prepared in parallel ($g_{∥}^{\left(2\right)}$, blue) and perpendicular ($g_{⊥}^{\left(2\right)}$, orange) polarization, under CW resonant excitation. (**C** and **D**) Normalized photon coincidences ($g_{∥}^{\left(2\right)}$, $g_{⊥}^{\left(2\right)}$) for input photons from QD1 (C) and both QDs (D), under pulsed resonant excitation. The measurement in (A) and (B) and in (C) and (D) is done at *P*/*P*_sat_ ≈ 0.1 and pulse area of π/2, respectively. Bottom: The corresponding interference visibility *V*_HOM_, calculated from the expression $1-g_{∥}^{\left(2\right)}/g_{⊥}^{\left(2\right)}$ for each case.

Figure 4 (A and B) shows the results of the experiment for the emission from only QD1 and from both QDs when degenerate, respectively, under CW resonant driving. The temporal postselected indistinguishability for QD1 yields *V*_HOM_(0) → 1, as expected for an individual emitter. However, for cooperative emission from both QDs, we observe a maximum visibility of *V*_HOM_(0) ≈ 0.5. This result signals a significant change in emitted light, from being antibunched (sub-Poissonian) for a single QD to having Poissonian-like statistics due to cooperative emission. Notably, the 1/*e* width of *V*_HOM_, which gives the coherence time of the emission, for the cooperative case (≈1.15 ns) is comparable to the single-emitter case (≈1.34 ns). Using the coherence time window (CTW) (*41*–*43*), i.e., the integrated area of *V*_HOM_, as the figure of merit, we find that the CTW for the single-emitter case [1.37 (1) ns] is more than twice that of the cooperative emission [0.53 (1) ns], reflecting the difference for each case in both the *V*_HOM_(0) values and 1/*e* widths. Beyond the time-averaged picture, Fig. 4 (C and D) shows the result of HOM-type interference for the emission from QD1 and both QDs under pulsed resonant driving, respectively. For QD1, we observe *V*_HOM_ = 0.40 (1) for the non-postselected (10-ns integration window) visibility and a maximum postselected (0.1-ns integration window) visibility of *V*_HOM_ = 0.79 (10). These results indicate partial distinguishability between successively emitted photons from QD1. Similarly, compared to *V*_HOM_ = 0.5 obtained from the CW measurement and *V*_HOM_ = 0.37 (2) in the 0.1-ns integration window, degradation in the non-postselected visibility for the cooperative emission is observed: *V*_HOM_ = 0.20 (1) within a 10-ns integration window.

## DISCUSSION

In summary, we coherently control and probe two QDs coherently coupled by a photon-mediated interaction enabled by the indistinguishability of their photon wave packets and path erasure of their spatial positions. We find that both *g*^(2)^(τ) and lifetime measurements are necessary to fully describe the nature of the coherent coupling; *g*^(2)^(τ) on its own is not a sufficient witness of superradiance. Compared to incoherent driving, we demonstrate that coherent driving enables both increased emitter indistinguishability and control of the populations on the Dicke ladder. We find that after emission of the first photon, both emitters are in an entangled state, as signified by *g*^(2)^(0) → 1. Furthermore, we show that *g*^(2)^(τ) is a sensitive probe of the dephasing of the emitters. For coherent driving, the dephasing likely originates from emitter coupling to the solid-state environment [phonon-induced dephasing (*43*–*45*) or charge and spin noise (*46*)], which can be at least partially mitigated with improved device quality, choice of charge state, and Purcell enhancement. Last, our measurements reveal Poissonian-like statistics of the cooperative emission, analogous to a weak coherent state from an attenuated laser (*35*). This result demonstrates the coherence of the cooperative emission and evokes the analogy between cooperative emission, in which indistinguishable atomic dipoles are locked in-phase, and lasing (*2*). Exciting prospects would be to increase the number of cooperative emitters using scalable approaches and include spin control of the coherently coupled emitters. This would create more intermediate states on the Dicke ladder, allow better understanding of the collective behavior of interacting many-body systems, and provide a potential route to tailor the cooperative behavior and harness collective light-matter interaction effects for photon-mediated applications.

## MATERIALS AND METHODS

### Sample structure

The experiments are performed on self-assembled InGaAs QDs embedded in a GaAs Schottky diode for deterministic charge control via the applied gate voltage. The gate voltage induces DC Stark shift and shifts the emission wavelength of the emitter, with a typical linear response of 1 to 2 μeV/mV. A broadband planar cavity and glass SIL (not shown in Fig. 1A) are used to enhance the photon extraction efficiency (*37*). Specific details of the sample heterostructure are described in (*47*).

### Spectroscopy setup

The sample is kept at a temperature of 4 K in a closed-cycle helium flow cryostat. A polarization-based dark-field confocal microscope is used to excite and collect the resonance fluorescence from the QDs while suppressing the scattered laser light (typical extinction ratio ≈10^7^) (*48*). The photons are sent to either a spectrometer (resolution of 40 μeV) equipped with a liquid nitrogen–cooled charge-coupled device or a pair of superconducting nanowire single-photon detectors with a nominal detection efficiency of 90% at 950 nm and a time jitter of ≈100 ps. For HOM measurements, we spectrally filter the phonon sideband using a grating filter with an FWHM of 120 (1) μeV and 45 to 50% fiber-to-fiber transmission efficiency. The CW experiments are done with a narrowband (submegahertz linewidth) diode laser, while the pulsed driving is achieved with a mode-locked femtosecond laser, stretched to ~40-ps pulse width, with an 80-MHz repetition rate. Emitter lifetime and photon correlations (HBT and HOM) are made with a time-correlated single-photon counting system.

### Coincidence measurements under cooperative emission

Photon coincidences provide a useful tool to characterize cooperative emission. To derive the signatures of cooperative emission, we consider the light-matter interaction between two QDs described as two-level systems, where ∣*g_i_*⟩ and ∣*e_i_*⟩ are ground and excited states of the *i*th QD. Introducing the QD operators $\sigma_{i}^{+}=|e_{i}\rangle \langle g_{i}|$ and $\sigma_{i}^{-}=|g_{i}\rangle \langle e_{i}|$ as well as photon creation and annihilation operators $a_{k}^{†}$ and *a*_**k**_, the light-matter interacting Hamiltonian is

$$H_{I}=\sum_{k}\hbar g[(e^{ik\cdot r/2}\sigma_{1}^{-}+e^{-ik\cdot r/2}\sigma_{2}^{-})a_{k}^{†}+h.c.]$$ (4)

If the two QDs are fully distinguishable, then the emission of photons can be described by nondegenerate perturbation theory. The signal registered at the detectors is then simply a sum of the individual detection events

$$g_{\text{indep}.}^{\left(2\right)}(t,\tau )=\frac{\sum_{i,j=1}^{2}\langle \sigma_{i}^{+}(t)\sigma_{j}^{+}(t+\tau )\sigma_{j}^{-}(t+\tau )\sigma_{i}^{-}(t)\rangle }{\sum_{i,j=1}^{2}\langle \sigma_{i}^{+}(t)\sigma_{i}^{-}(t)\rangle \langle \sigma_{j}^{+}(t+\tau )\sigma_{j}^{-}(t+\tau )\rangle }$$ (5)

Following the emission of a photon, one QD will be in its ground state and can thus no longer contribute to the immediate emission of a second photon, i.e., $\sigma_{i}^{-}\sigma_{i}^{-}=0$. Hence, only emission processes from different emitters *i* ≠ *j* contribute to the numerator, whereas all emission processes contribute to the denominator. For initially uncorrelated and distinguishable QDs with exciton populations *n*_1_ and *n*_2_ at time *t*, the zero-delay coincidences are therefore

$$g_{\text{indep}.}^{\left(2\right)}(t,0)=\frac{2n_{1}n_{2}}{{\left(n_{1}+n_{2}\right)}^{2}}\leq \frac{1}{2}$$ (6)

where the limit 1/2 is reached for *n*_1_ = *n*_2_.

However, when the QDs are indistinguishable, both QDs feed into the same electromagnetic field modes, and a signal measured at the detector can no longer resolve from which QD a detected photon originated. To describe the measurement process, it is instructive to rewrite the light-matter interaction

$$H_{I}=\sum_{k}\hbar g\sqrt{2}(\sigma_{k}^{-}a_{k}^{†}+\sigma_{k}^{+}a_{k})$$ (7)

in terms of operators $\sigma_{k}^{\pm }=(e^{ik\cdot r/2}\sigma_{1}^{\pm }+e^{-ik\cdot r/2}\sigma_{2}^{\pm })/\sqrt{2}$, which describe the dipole operator responsible for driving the photon mode with wave vector **k**.

In our setup, we predominantly detect photons emitted perpendicular to the plane containing the QDs, i.e., photons with wave vectors **k** ≈ **k**_0_, where **k**_0_ is a reference wave vector with **k**_0_ ⊥ **r** and modulus ∣**k**_0_∣ matching the common transition frequency of the QDs. In this situation, *e*^±*i***k** · **r**^ ≈ 1 so that the photon modes picked up by the detector are driven by the dipole operators $\sigma_{S}^{\pm }=(\sigma_{1}^{\pm }+\sigma_{2}^{\pm })/\sqrt{2}$, describing transitions through the symmetric Dicke state $|\psi_{S}\rangle =(|e_{1},g_{2}\rangle +|g_{1},e_{2}\rangle )/\sqrt{2}$ (cf. Fig. 1C).

In particular, the second-order coherence takes the form

$$g_{\text{coop}}^{\left(2\right)}.(t,\tau )=\frac{\langle \sigma_{S}^{+}(t)\sigma_{S}^{+}(t+\tau )\sigma_{S}^{-}(t+\tau )\sigma_{S}^{-}(t)\rangle }{\langle \sigma_{S}^{+}(t)\sigma_{S}^{-}(t)\rangle \langle \sigma_{S}^{+}(t+\tau )\sigma_{S}^{-}(t+\tau )\rangle }$$ (8)

With occupation *n*_*e*_1_, *e*_2__ of the doubly excited state ∣*e*_1_, *e*_2_⟩ and occupations *n_S_* of the symmetric state ∣ψ*_S_*⟩, the zero-delay coincidences are

$$g_{\text{coop}.}^{\left(2\right)}(t,0)=\frac{n_{e_{1},e_{2}}}{{\left(n_{e_{1},e_{2}}+n_{S}\right)}^{2}}$$ (9)

which, for initially uncorrelated states with equal exciton populations, results in $g_{\text{coop}.(t,0)=1}^{\left(2\right)}$.

The decisive difference to the situation of independent emitters is that the emission of the first photon does not impede the emission of a second photon. Instead, both emitters cooperatively contribute to both emission processes, leading to zero-delay coincidences that exceed the limit of ^1^/_2_ for independent emitters.

A minimal model for calculating the time dependence of *g*^(2)^(*t*, τ) is given in section S1.

## Notes

** Correction (8 April 2022):** During production, three typographical errors were introduced in the equations on page four of the Results section. The equations have been corrected in the PDF and HTML.

## Supplementary Materials

### This PDF file includes:

Sections S1 to S5 Figs. S1 to S5 Tables S1 and S2
