# Supplementary material for: Coherence in cooperative photon emission from indistinguishable quantum emitters
Source: Sci Adv. 2022 Mar 18;8(11):eabm8171. doi: 10.1126/sciadv.abm8171 (PMC8932659; doi:10.1126/sciadv.abm8171)
Supplement: Supplementary file 1 — Sections S1 to S5 Figs. S1 to S5 Tables S1 and S2 [file sciadv.abm8171_sm.pdf]

Science Advances
AAAS

Supplementary Materials for

Coherence in cooperative photon emission from indistinguishable quantum emitters

Zhe Xian Koong*, Moritz Cygorek, Eleanor Scerri, Ted S. Santana, Suk In Park, Jin Dong Song, Erik M. Gauger, Brian D. Gerardot*

*Corresponding author. Email: zk49@hw.ac.uk (Z.X.K.); b.d.gerardot@hw.ac.uk (B.D.G.)

Published 18 March 2022, *Sci. Adv.* **8**, eabm8171 (2022)
DOI: 10.1126/sciadv.abm8171

The PDF file includes:

Sections S1 to S5
Figs. S1 to S5
Tables S1 and S2

Other Supplementary Material for this manuscript includes the following:

Dataset

S1. MINIMAL MODEL FOR $g^{(2)}(\tau)$

Emission model:	$\partial\rho/\partial t =$	$G^{(2)}(\tau) =$
**single**	$\gamma\mathcal{L}\big[\sigma_1^-\big](\rho) + \gamma_p\mathcal{L}\big[\sigma_1^+\big](\rho) + \gamma_d\mathcal{L}\big[\sigma_1^+\sigma_1^-\big](\rho)$	$\langle\sigma_1^+\sigma_1^+(\tau)\sigma_1^-(\tau)\sigma_1^-\rangle$
**independent**	$\gamma\mathcal{L}\big[\sigma_1^-\big](\rho) + \gamma_p\mathcal{L}\big[\sigma_1^+\big](\rho) + \gamma_d\mathcal{L}\big[\sigma_1^+\sigma_1^-\big](\rho)$ $+\gamma\mathcal{L}\big[\sigma_2^-\big](\rho) + \gamma_p\mathcal{L}\big[\sigma_2^+\big](\rho) + \gamma_d\mathcal{L}\big[\sigma_2^+\sigma_2^-\big](\rho)$	$\sum_{i,j=1,2}\langle\sigma_i^+\sigma_j^+(\tau)\sigma_j^-(\tau)\sigma_i^-\rangle$
**cooperative**	$\gamma\mathcal{L}\big[\sigma_1^-\big](\rho) + \gamma_p\mathcal{L}\big[\sigma_1^+\big](\rho) + \gamma_d\mathcal{L}\big[\sigma_1^+\sigma_1^-\big](\rho)$ $+\gamma\mathcal{L}\big[\sigma_2^-\big](\rho) + \gamma_p\mathcal{L}\big[\sigma_2^+\big](\rho) + \gamma_d\mathcal{L}\big[\sigma_2^+\sigma_2^-\big](\rho)$	$\langle\sigma_S^+\sigma_S^+(\tau)\sigma_S^-(\tau)\sigma_S^-\rangle$
**superradiant**	$\Gamma\mathcal{L}\big[\sigma_S^-\big](\rho) + \gamma_p\mathcal{L}\big[\sigma_1^+\big](\rho) + \gamma_d\mathcal{L}\big[\sigma_1^+\sigma_1^-\big](\rho)$ $+\gamma_p\mathcal{L}\big[\sigma_2^+\big](\rho) + \gamma_d\mathcal{L}\big[\sigma_2^+\sigma_2^-\big](\rho)$	$\langle\sigma_S^+\sigma_S^+(\tau)\sigma_S^-(\tau)\sigma_S^-\rangle$

TABLE I. Equations of motion and expressions for the second order (intensity) correlation function $G^{(2)}$ for the cases of a single emitter, independent emitters, selectively measured emitters, and superradiantly coupled emitters.

Here, we formulate a minimal model to describe the time delay dependence of $g^{(2)}(\tau)$ for single emitters, independent emitters, selectively measured coherently coupled emitters, and superradiantly coupled emitters. To this end, we need to calculate two-time correlation functions of the form

$$\begin{aligned} &\langle a^\dagger_{\mathbf{k}_1}(t_1)a^\dagger_{\mathbf{k}_2}(t_1+\tau)a_{\mathbf{k}_2}(t_1+\tau)a_{\mathbf{k}_1}(t_1)\rangle \\ &= \mathrm{Tr}\Big[a_{\mathbf{k}_2}(t_1+\tau)a_{\mathbf{k}_1}(t_1)\rho(0)a^\dagger_{\mathbf{k}_1}(t_1)a^\dagger_{\mathbf{k}_2}(t_1+\tau)\Big] \\ &= \mathrm{Tr}\Big[a_{\mathbf{k}_2}U(\tau)\big(a_{\mathbf{k}_1}\rho(t_1)a^\dagger_{\mathbf{k}_1}\big)U^\dagger(\tau)a^\dagger_{\mathbf{k}_2}\Big]. \end{aligned} \tag{S1}$$

This can be achieved by propagating the density matrix ρ using the time evolution operator $U(t)$ up to time t_1, applying the photon operators $a^{(\dagger)}_{\mathbf{k}_1}$, and propagating the resulting non-normalized pseudo density matrix for a time τ, before applying the second set of photon operators $a^{(\dagger)}_{\mathbf{k}_2}$. The photon operators $a_{\mathbf{k}}$ are replaced by system operators σ_i^- or σ_S^- depending on the situation (cf. main text).

In our minimal model, we describe the time evolution before and in between the photon detection events using incoherent pump, decay, and dephasing rates. Because the selective measurement or superradiant decay introduces coherence between excited states in the two emitters, we use master equations of Lindblad form to correctly capture the corresponding off-diagonal elements in the density matrix, replacing the time evolution operators $U(t)$ in Eq. (S1) by the corresponding propagator in Liouville space.

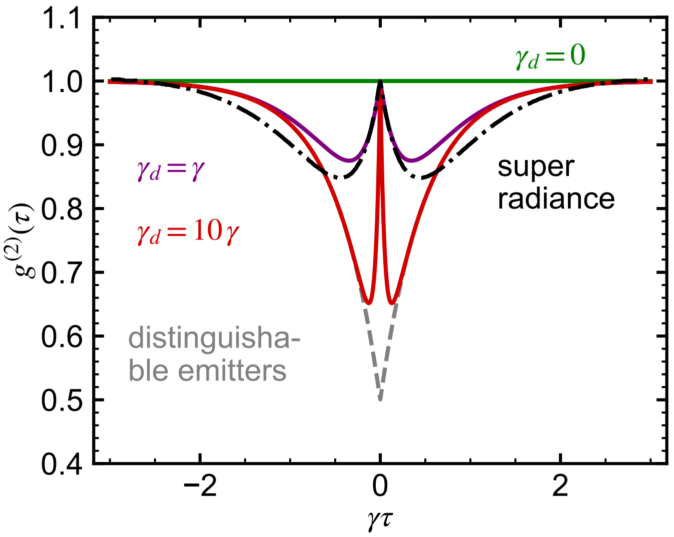

FIG. S1. **Second-order correlation function ($g^{(2)}(\tau)$) for selective measurement and superradiant case.** $g^{(2)}(\tau)$ as a function of detection time delay, τ shows similar anti-dip feature around $\tau = 0$ as a result of emission from two indistinguishable emitters, under the selective measurement ($\gamma_d \neq 0$, solid lines) and superradiance (dashed-dotted line). In the absence of pure dephasing ($\gamma_d = 0$, solid line), $g^{(2)}(\tau) = 1$ at all τ. For the case of two distinguishable emitters (dashed line), this feature disappears and the $g^{(2)}(0) = 0.5$ instead.

Using the Lindblad superoperator

$$\mathcal{L}[A](\rho) = A\rho A^\dagger - \tfrac{1}{2}\left(A^\dagger A\rho + \rho A^\dagger A\right), \tag{S2}$$

the time evolution of a single incoherently pumped emitter can be written as

$$\frac{\partial}{\partial t}\rho = \gamma\mathcal{L}\left[\sigma_1^-\right](\rho) + \gamma_p\mathcal{L}\left[\sigma_1^+\right](\rho) + \gamma_d\mathcal{L}\left[\sigma_1^+\sigma_1^-\right](\rho), \tag{S3}$$

where the first term describes radiative decay with rate γ, the second term accounts for incoherent pumping with rate γ_p, and the third term phenomenologically describes pure-dephasing with a dephasing rate γ_d. The latter has the effect of reducing coherence between the ground $|g_1\rangle$ and excited state $|e_1\rangle$ and originates microscopically, e.g., from interactions with the phonon environment.

The equations of motion for two independent emitters are obtained by adding to the equations of motion for a single emitter the same set of equations but with operators $\sigma_1^\pm$ replaced by $\sigma_2^\pm$. In the case of superradiance, we assume the pumping of both emitters to be independent, but radiative decay involves transitions through the symmetric state

$$\frac{\partial}{\partial t}\rho = \Gamma\mathcal{L}\left[\sigma_S^-\right](\rho) + \gamma_p\mathcal{L}\left[\sigma_1^+\right](\rho) + \gamma_p\mathcal{L}\left[\sigma_2^+\right](\rho), \tag{S4}$$

where for ideal (no pure dephasing) superradiance $\Gamma = 2\gamma$. The respective equations of motion for the different cases together with the corresponding expressions for the second order coherence are listed in Table I. With these results, the numerical calculation of the second order correlation function is straightforward. If not stated otherwise, we use parameters $\gamma_p = \gamma$ and $\gamma_d = 0$, where $\gamma = 1$ defines the unit of time.

S2. RELATION TO SUPERRADIANCE

To further clarify the nature of cooperative emission in absence of superradiance, we now describe the link to superradiance on the one hand and to independent emission on the other hand in terms of wave vector selectivity of coincidence measurements.

Consider two detectors that predominantly register photons at wave vectors $\mathbf{k}_0^{(1)}$ and $\mathbf{k}_0^{(2)}$, respectively. Then, a first photon detection event described by the operators $\sigma^\pm_{\mathbf{k}_0^{(1)}}$ from an initially doubly occupied two-emitter system

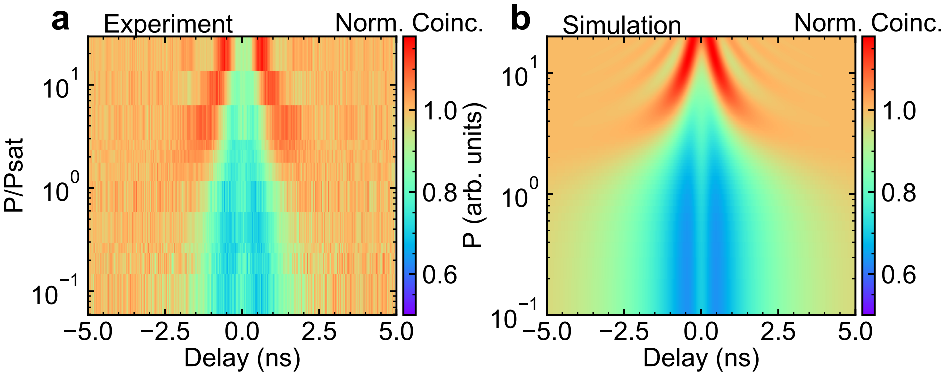

FIG. S2. **Power dependence of the continuous wave resonant excitation of two QDs tuned into resonance.** A comparison of the experimental **a** and simulated data **b** for $g^{(2)}(\tau)$ as a function the ratio of the excitation power, P. The excitation power in the experimental data is normalized by the saturation power, Psat.

leads to a collapse of the emitter state to $|\psi_{\mathbf{k}_0^{(1)}}\rangle$. The conditional probability of detecting a second photon by detector 2 as described by operators $\sigma^{\pm}_{\mathbf{k}_0^{(2)}}$ is given by

$$G^{(2)}_{\mathbf{k}_0^{(1)},\mathbf{k}_0^{(2)}}(t,0) = \langle \sigma^+_{\mathbf{k}_0^{(1)}} \sigma^+_{\mathbf{k}_0^{(2)}} \sigma^-_{\mathbf{k}_0^{(2)}} \sigma^-_{\mathbf{k}_0^{(1)}} \rangle = \langle \psi_{\mathbf{k}_0^{(1)}} | \sigma^+_{\mathbf{k}_0^{(2)}} \sigma^-_{\mathbf{k}_0^{(2)}} | \psi_{\mathbf{k}_0^{(1)}} \rangle = |\langle \psi_{\mathbf{k}_0^{(2)}} | \psi_{\mathbf{k}_0^{(1)}} \rangle|^2 = \frac{1}{2}\Big[1 + \cos\big((\mathbf{k}_0^{(2)} - \mathbf{k}_0^{(1)}) \cdot \mathbf{r}\big)\Big], \tag{S5}$$

which shows a strong dependence of photon coincidences on the relative emission directions of the first and the second photon. Eq. (S5) can be interpreted as follows: The first photon measurement process prepares the system in a state whose oscillator strength for the emission of a second photon strongly depends on the emission direction $\mathbf{k}_0^{(2)}$, encoded in the relative phase $e^{i\mathbf{k}_0^{(1)}\cdot\mathbf{r}}$ between states $|e_1, g_2\rangle$ and $|g_1, e_2\rangle$ in the correlated intermediate state $|\psi_{\mathbf{k}_0^{(1)}}\rangle$.

In the superradiant regime $\mathbf{k}_0^{(i)} \cdot \mathbf{r} \approx 0$, so that the states involved in the transitions have identical phases $|\psi_{\mathbf{k}_0^{(2)}}\rangle = |\psi_{\mathbf{k}_0^{(1)}}\rangle = |\psi_S\rangle$, leading to a perfect overlap $|\langle \psi_{\mathbf{k}_0^{(2)}} | \psi_{\mathbf{k}_0^{(1)}} \rangle|^2 = 1$ and hence a maximal value of $G^{(2)}_{\mathbf{k}_0^{(1)},\mathbf{k}_0^{(2)}}(t,0) = 1$, irrespective of the emission directions defined by $\mathbf{k}_0^{(1)}$ and $\mathbf{k}_0^{(2)}$.

By contrast, if the distance r is larger than the wavelength of the emitted light and all photons are collected, i.e. no particular wave vectors are selected, then the relative phases $e^{i\mathbf{k}_0^{(i)}\cdot\mathbf{r}}$ are random, resulting in destructive interference of the cosine term in Eq. (S5). This leads to exactly half as many photon coincidences as in the superradiant regime, analogous to what we obtain from simply considering independent emitters.

Finally, in our confocal microscope, we predominantly detect photons emitted perpendicular to the sample plane containing the QDs, which implies that all detected photons have similar wave vectors $\mathbf{k}_0^{(2)} = \mathbf{k}_0^{(1)}$. Therefore, with $\cos\big((\mathbf{k}_0^{(2)} - \mathbf{k}_0^{(1)}) \cdot \mathbf{r}\big) = 1$, the same enhancement of $G^{(2)}(t,0) = 1$ is found as in the superradiant regime. This remains true even though the distance between the emitters may be significantly larger than the wavelength of the emitted light. In both of the 'cooperative' cases, i.e. superradiance as well as the present case of cooperative emission without superradiance, the enhancement of photon coincidences with respect to independent emitters can be attributed to the perfect matching between intermediate states involved in the emission processes of the first and the second photon.

S3. COOPERATIVE EMISSION UNDER CONTINUOUS WAVE COHERENT DRIVING: POWER DEPENDENCE

Figure S2 shows the theory-experiment comparison of the power dependence of the $g^{(2)}(\tau)$ data under continuous wave coherent (resonant) excitation of two indistinguishable quantum dots. We observe Rabi oscillations as we increase the driving strength beyond the saturation power, Psat. The experimental data agrees qualitatively with the simulated data obtained using the theoretical model defined in Section S1 and the emitter parameters (e.g. spontaneous emission rate and dephasing rate), extracted from the experimental data in Figure 2d in the main manuscript.

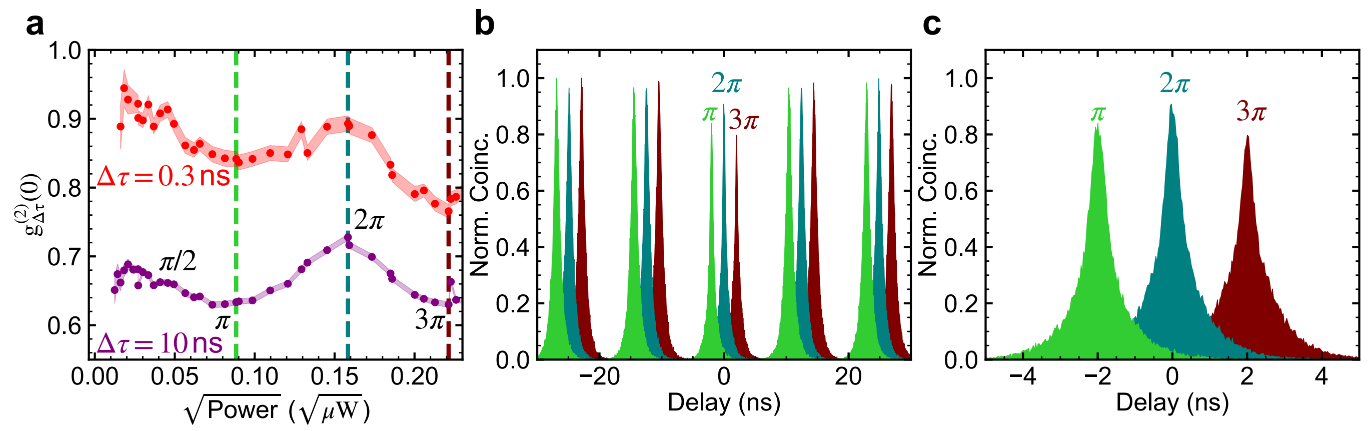

FIG. S3. **Power dependence of the pulsed resonant excitation of two QDs tuned into resonance.** **a** Normalized zero-delay coincidences from the HBT measurement, $g^{(2)}_{\Delta\tau}(0)$ under resonant excitation, as a function of excitation power, within 0.3 ns (red) and 10 ns (purple) integration windows. **b, c** Coincidence histogram at a pulse area of π, 2π and 3π within 60 (**b**) and 10 ns (**c**) coincidence window. The data for π and 3π are shifted temporally by -2 and 2 ns, respectively.

S4. COOPERATIVE EMISSION UNDER PULSED COHERENT DRIVING: POWER DEPENDENCE

Figure S3 shows the power dependence of the $g^{(2)}(\tau)$ data under pulsed coherent (resonant) excitation of two indistinguishable quantum dots. We demonstrate coherent control and the ability to manipulate the populations on the Dicke ladder as we vary the excitation power of the resonant pulse and observe that the $g^{(2)}_{\Delta\tau=0.3\,\mathrm{ns}}(0)$ ($g^{(2)}_{\Delta\tau=10\,\mathrm{ns}}(0)$) value oscillates with pulse area: a maximum of 0.90 (0.73) at 2π pulse area and a value of 0.84 (0.63) at π pulse area. The power dependence and the coincidence histograms that correspond to the three example pulse areas are depicted in Figure. S3(a) and S3(b, c), respectively. This result suggests that the measured $g^{(2)}_{\Delta\tau}(0)$ depends on the population of the bright, symmetric Dicke state: a minimal $g^{(2)}_{\Delta\tau}(0)$ value at pulse areas of odd multiples of π, where the system remains approximately in the ground state and a maximal $g^{(2)}_{\Delta\tau}(0)$ value at pulse areas of even multiples of π, where the system is close to the doubly-excited state which then collapses onto the symmetric Dicke state following the first photon detection event. The contribution from the detection of any additional unintended photon, e.g. due to stray light or an increase of populations in the bright, symmetric Dicke state due to re-excitation during the pulse result in a increase in correlation and a hence a higher $g^{(2)}_{\Delta\tau}(0)$. Conversely, the possibility of the re-excitation of the doubly-excited state along with the subsequent decay to the ground state during the remainder of the pulse result to a lower $g^{(2)}_{\Delta\tau}(0)$. Full interpretation and modeling of this trend require the sufficient knowledge of multiple key parameters, including, but not limited to re-excitation rate [47], excitation-induced dephasing rate [44], and signal-to-background ratio due to laser leakage as a function of driving power.

S5. COOPERATIVE EMISSION UNDER PULSED INCOHERENT DRIVING

Please refer to the captions of Figure S4 for additional data on the spectroscopy of the emission from two QDs under pulsed incoherent, non-resonant (phonon-assisted) excitation.

S6. CORRECTION TO THE DEGREE OF ENTANGLEMENT DUE TO NOISE AND DETECTION JITTER

In this section, we provide an analysis of the degree of entanglement for simultaneous detection of the emission from two indistinguishable emitters in the presence of the noise and detection jitter. As described in Section 8 of the Supplementary Material in Ref. [10], one can extract the ratio of noisy photons compared to single photons p_n extracted from the $g^{(2)}_{\mathrm{QD1}}(0)$ for individual QD emission, i.e.

$$g^{(2)}_{\mathrm{QD1}}(0) = \frac{p_n(2+p_n)}{(1+p_n)^2}. \qquad \text{(S6)}$$

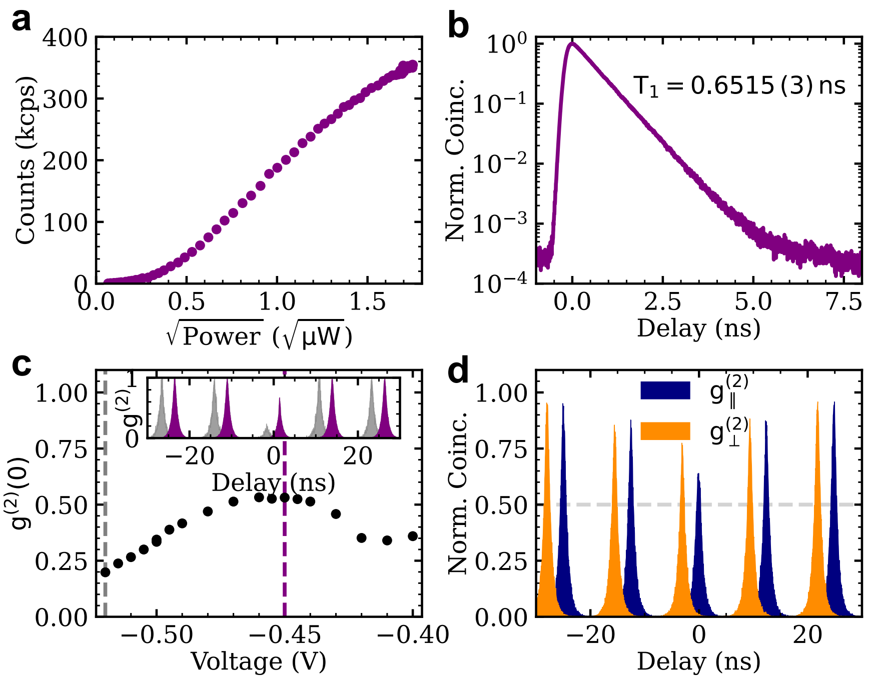

FIG. S4. **Pulsed non-resonant excitation of two QDs tuned into resonance.** **(a)** Emission count rates from two QDs tuned into resonance, as a function of excitation power at bias of -0.45 V. **(b)** Time-resolved emission profile of the cooperative emission at power of $0.58\,\mu$W. **(c)** Normalized zero-delay coincidences from the HBT measurement, $g^{(2)}(0)$ as a function of applied bias (excitation power = $0.58\,\mu$W). Inset shows the normalized coincidences from the HBT measurement at -0.52 V ($\delta \approx 95\,\mu$eV, grey) and -0.45 V ($\delta \approx 0\,\mu$eV, purple). **(d)** Two-photon interference between subsequently emitted photons, separated by 12.44 ns, for the case where input photons, from both QDs tuned into the resonance ($\delta = 0$) are prepared in parallel ($g^{(2)}_{\parallel}$, blue) and perpendicular ($g^{(2)}_{\perp}$, orange) polarization. The non-post-selected (post-select within 100 ps window) interference visibility for this measurement is 0.041 (0.212). All measurements are done with 14 ps excitation pulses, frequency detuned by ≈ 0.4 meV from the two-dot-degeneracy at $\lambda = 971.169\,$nm.

Using this information, we can obtain the degree of entanglement, given by the fidelity to the maximally entangled symmetric Dicke state $(1/\sqrt{2}\,(|e_1, g_2\rangle + |g_1, e_2\rangle))$, $\mathcal{F}$ using

$$\mathcal{F} \geq \frac{(g^{(2)}(0)(1+p_n)^2 - p_n(2+p_n))^2}{g^{(2)}(0)(1+p_n)^2}. \tag{S7}$$

In the noiseless limit (i.e. $p_n = 0$), this is given solely by the measured (normalized) coincidences at the zero-delay in the second-order intensity correlation function $g^{(2)}(0)$. The presence of surplus "noisy" photons in the signal (which may originate from further emitters or the excitation laser) and the timing jitter in the detection setup result in a decrease in the entanglement fidelity $\mathcal{F}$. To account for both effects, we extract the ratio of noisy photons in the single photon stream from an intensity correlation measurement with only a single emitter. We fit the experimental data in Figure 2d and 3d by convolving the theoretical expressions in Eq. 1 and 2 with the independently measured detection timing jitter (full-width-at-half-maximum of 0.240 ns) and compare them with the corresponding deconvolved fits. The fits, along with the experimental data are depicted in Figure S5. We summarise the results, i.e. the change in the $g^{(2)}(0)$ value with respect to each imperfections in Table II.

It is important to note that after deconvolution, the zero-delay peak (pulsed excitation) and the anti-dip (continuous wave excitation) have a maximum close to one, as expected from the ideal noise-less case for the emission from two indistinguishable emitters. This shows that the maximum of the zero-delay peak is largely determined by detector jitter and hence is inadequate to quantify the entanglement of the emitters. This is analogous to the interpretation of the zero-delay anti-bunching dip in the Hong-Ou-Mandel interference measurement of subsequently emitted single photons; please refer to Refs. [41-43] for more details. Instead, the integration over the full zero-delay peak within a large integration window ($g^{(2)}_{\Delta\tau}$) gives information about the dephasing of the emitted photons, independent of the detector jitter, and hence is more suitable and useful to characterise the entanglement of the emitters.

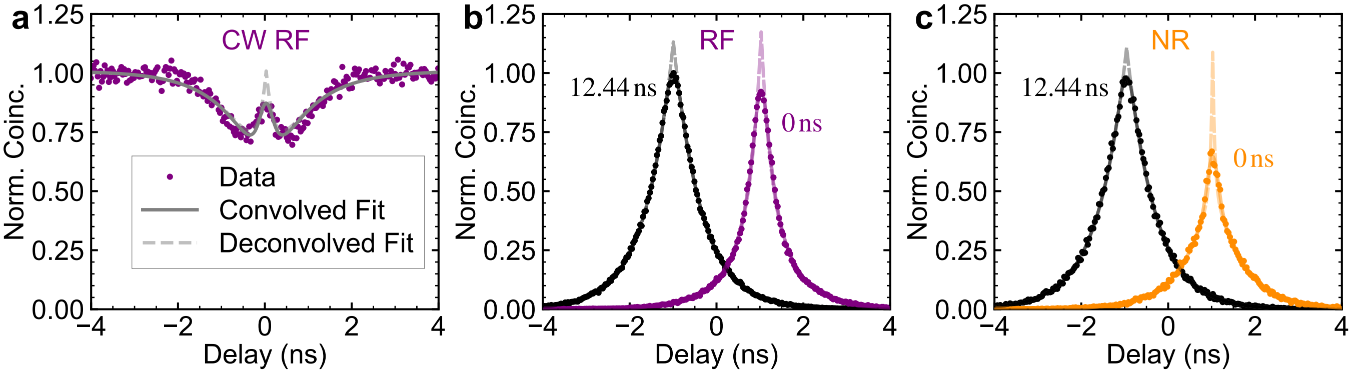

FIG. S5. **Comparison between experimental data, the convolved and deconvolved fit for the second order intensity correlation measurement with two indistinguishable emitters.** Normalized coincidence (to the raw coincidences at longer delays of $\approx 10\,\mu$s) under continuous wave resonant (CW RF, **a**), pulsed resonant (RF, **b**) and non-resonant excitation (NR, **c**). Convolved and deconvolved fits are given by the solid and dashed lines, respectively. Experimental data and the convolved fits are the same as in Figure 2d (CW) and 3d (Pulsed). The uncorrelated side peak at 12.44 ns and the correlated zero-delay peak are shifted by -1 and 1 ns, respectively.

TABLE II. The degree of entanglement, indicated by the normalized zero-delay coincidences $g^{(2)}(0)$ from two degenerate QDs under CW and pulsed resonant (RF) and non-resonant (NR) excitation in the presence of noise, given by the ratio of noisy photons in the QD signal p_n and the detection timing jitter (modelled as a Gaussian with a full-width-at-half-maximum of 0.240 ns). The correction to this quantity, given by the fidelity to symmetric Dicke state $\mathcal{F}$, as a result of the presence of noisy photons is estimated using the expression in Sec. 8 of the Supplementary Material in Ref. [10] using the extracted ratio of noisy photons to QD signal, p_n in the single emitter case. For the case with pulsed measurements, the values reported here are extracted from the coincidence histogram with integration windows of 10 ns ($g^{(2)}_{\Delta\tau=10\,\mathrm{ns}}(0)$). The values with temporal post-selection of $\leq$ 0.1 ns ($g^{(2)}_{\Delta\tau\leq 0.1\,\mathrm{ns}}(0)$) are listed in parenthesis.

	CW	Pulsed	
	RF^a	RF^b	NR^c
Measured QD1 $g^{(2)}_{\mathrm{QD1}}(0)$	0.06	0.05 (0.05)	-
Ratio of noisy photons p_n	0.031	0.026 (0.026)	-
Measured QD1+QD2 $g^{(2)}(0)$	0.87	0.67 (0.93)	0.56 (0.65)
Deconvolved $g^{(2)}_{\mathrm{Deconv.}}(0)$	1	0.68 (1.04)	0.54 (0.98)
$\mathcal{F}$ ^{d}	0.80	0.60 (0.88)	0.56 (0.65)
$\mathcal{F}^{\mathrm{Deconv.}\ e}$	0.94	0.61 (0.99)	0.54 (0.98)

[a] Data taken at P $\sim 0.3\,\mathrm{P_{sat}}$ [b] Data taken at $\pi/2$-pulse [c] Data taken at saturation. [d] Correction to the $g^{(2)}(0)$.
[e] Correction to the $g^{(2)}_{\mathrm{Deconv.}}(0)$.
